# Spatial distribution of the sibling species of A*nopheles gambiae* sensu lato (Diptera: Culicidae) and malaria prevalence in Bayelsa State, Nigeria

**DOI:** 10.1186/1756-3305-7-32

**Published:** 2014-01-17

**Authors:** Amawulu Ebenezer, Aline Edith Mekeu Noutcha, Peter I Agi, Samuel N Okiwelu, Thomas Commander

**Affiliations:** 1 Department of Animal and Environmental Biology, University of Port-Harcourt, Port-Harcourt, Nigeria; 2 Department of Biology, Isaac Jasper Boro College of Education, Bayelsa State, Nigeria; 3 Department of Biological Sciences, Niger Delta University, Wilberforce Island, Bayelsa State, Nigeria

**Keywords:** *An. gambiae s.l*, Sibling species, Malaria prevalence, GIS, Distribution, Bayelsa- State

## Abstract

**Background:**

Much of the confusing ecophenotypic plasticity of *Anopheles gambiae sensu lato* is attributable to the differential biological traits of the sibling species, with their heterogeneous geographical distribution, behavioral dissimilarities and divergent population dynamics. These differences are critical to their roles in malaria transmission. Studies were, therefore, undertaken on the spatial distribution of these species and malaria prevalence rates in Bayelsa State, September, 2008-August 2010.

**Methods:**

Mosquito sampling was in 7 towns/villages in 7 Local Government Areas (LGAs) in 3 eco-vegetational zones: Fresh Water Swamp Forest (FWSF): Sagbama, Yenagoa, Kolokuma-Opokuma LGAs; Brackish Water Swamp Forest (BWSF): Ogbia, Ekeremor, Southern Ijaw LGAs; Mangrove Water Forest (MWF): Nembe LGA. Adults were collected twice quarterly by the Pyrethrum Spray Catch (PSC) technique. *Anopheles* was separated morphologically and the sibling species PCR- identified. Simultaneously, malaria prevalence rates were calculated from data obtained by the examination of blood smears from consenting individuals at hospitals/clinics*.*

**Results:**

*An. gambiae s.s.* was dominant across the 3-eco-vegetational zones. Spatial distribution analyses by cell count and nearest neighbor techniques indicated a tendency to clustering of species. *An. gambiae s.s.* and *An. arabiensis* clustered in Ekeremor LGA while these 2 species and *An. melas* aggregated in Nembe. The gonotrophic (physiological) status examination revealed that 34.3, 23.5, 23.1 and 18.4% of the population were fed, unfed, gravid and half gravid respectively. The highest malaria prevalence rates were obtained at Kolokuma-Opokuma and Nembe LGAs. Variation in prevalence rates among LGAs was significant (t = 5.976, df = 6, p-value = 0.002, p < 0.05). The highest prevalence rate was in the age group, 30-39 yrs, while the lowest prevalence was in the 0-9 yrs group.

**Conclusion:**

High malaria prevalence rates were associated with *An. gambiae s.s.* either in allopatry or sympatry across eco-vegetational zones. In areas where the sibling species clustered, they probably formed nidi for transmission. Socio-economic conditions might have contributed to reduced prevalence in Yenagoa, State Capital.

## Background

Much of the confusing eco-phenotypic plasticity of *An. gambiae s.l.* is the differential biological traits of the sibling species, their heterogeneous geographical distributions, behavioral dissimilarities and divergent population dynamics [1]. These differences are critical to the transmission of malaria in different zones of Nigeria [2-5]. Human malaria is caused by *Plasmodium* parasites and transmitted by female *Anopheles* mosquitoes. In Africa, the most efficient vectors are the *Anopheles gambiae* complex and *Anopheles funestus* group. *An. gambiae* is a complex of seven sibling species varying in their vectorial ability and ecological niche [6,7]. The sibling species are: the Freshwater *An. gambiae s.s.*, *An. arabiensis*, *An. quadrianulatus A*, *An. quadrianulatus B*; the salt water-breeding *An. melas* and *An. merus*, and *An. bwambae* found in hot springs in Uganda. The differences in the biology of the sibling species of *An. gambiae s.l.* have highlighted the need for mapping their spatial distribution and malaria prevalence patterns in order to enhance effective implementation of integrated control approaches [8]. Maps have been produced at continental and sub-regional scales [9]. The present study is aimed at developing a GIS- based overlay on the spatial patterns of PCR-identified sibling species of *An. gambiae* complex and *Plasmodium falciparum* malaria in Bayelsa State, Nigeria.

## Methods

### Study area

The study was conducted in 7 Local Government Areas (LGAs), Bayelsa State, Nigeria. Bayelsa State is located (5°22′E, 6°45′E and 4°15′N, 5°23′N) in the lower Delta plain formed during the Holocene of the quaternary period by the accumulation of sedimentary deposits [10]. The vegetation comprises three eco-vegetational zones: fresh water swamp forest, brackish water swamp forest and mangrove coastal water forest. The topography of study area is characterized by a maze of creeks and swamps criss-crossing the low-lying plain. The study LGAs were Yenagoa (4°53′N and 5°17′E), Sagbama (5°09′N and 6°14′E), Kolokuma-Opokuma (5°09′N and 6°14′E) in the fresh water swamp forest; Ogbia (4°53′N, 6°22′E), Southern Ijaw (4°07′N, 6°08′E) Ekeremor (5°02′N and 5°48′E) in the brackish water swamp forest and Nembe (4°27′N and 6°26′E) in the mangrove coastal water forest. All LGAs were rural, with the exception of semi-urban Yenagoa, the State capital. Many houses had traditional architectural design with mud walls and thatched roofs while few were built with blocks and roofed with corrugated iron sheets. The major occupations of the people were fishing, farming and petty trading.

### Ethical consideration

Before the commencement of the study, consent was obtained from the Ministry of Health, Bayelsa State, through the Primary Health Care (PHC) department, the village and household heads.

### Sample size

Sampling of the study population involved successive selection of new participants who presented at the Out-Patient Department of the 7 selected General Hospitals/ Clinics (Okolobiri, Olobiri, Sagbama, Amasoma, Ekeremor, and Kaiama) in each LGA until a sample size as described in Daniel [11] was obtained. These were individuals of all ages who had lived for at least 6 months and planned to stay for a further 6 months in the study areas. A total of 6321 individuals presented at the hospital, September, 2008- August, 2010.

### Blood sample collection

EDTA bottles were labeled following entry into the routine register with data on sex, occupation, and location of participants. A 2 ml-volume of intravenous blood was collected from each individual and transferred to a labeled EDTA bottle. Grease-free slides were labeled using patients’ details from the EDTA bottles. An aliquot of the blood was measured with a 1 ml-micropipette and dropped on a labeled slide to prepare thick and thin blood films following WHO standard procedures [12]. Preparations were air-dried and fixed with methanol for 30 seconds then stained with 4% Giemsa in phosphate buffer (7.2) for 30 minutes. Microscopy was used to examine the smears for the presence of malaria parasites under X1000 objective (Olympus, Japan) in oil immersion. Preliminary examination was carried out at the hospital where the blood was collected. The presence of malaria parasites in sexual and asexual stages was considered a positive diagnosis. The second and third examinations were at The Parasitology Research Laboratory, Department of Animal and Environmental Biology, University of Port Harcourt for quality assurance. Slides were reported as negative for malaria parasites after examining at least 50 fields and no parasites were detected. Prevalence rates were calculated.

### Mosquito collection

Collection of mosquitoes was undertaken in 7 villages/towns in the 7 LGAs. Their co-ordinates were obtained by geographic positioning system (GPS). The villages were randomly selected based on accessibility and availability of supporting staff. Selection of houses was based on their similarity in architectural designs. Six houses were used in each town/ village; these houses were utilized throughout the study. There were 1–2 rooms in each house.

Adult mosquitoes were collected by the Pyrethrum Spray Catch (PSC) method [13]), 0600-0730 hrs twice in each quarter, September, 2008- August, 2010. Selected rooms had at least one person sleeping overnight. Prior to spraying, the floors were covered with clean white sheets, outlets were closed and pyrethroid sprayed; the sheets were removed 15 min post-spray. Knocked down mosquitoes were picked up with pointed tip forceps and placed in labeled plastic cups. The gonotrophic (physiological) stages were determined as per WHO [14] and Noutcha and Anumudu [4]. Based on abdominal conditions, they were grouped as: unfed, fed, half gravid and gravid. Unfed females had a dark and flattened abdomen; fed had a dark red abdomen with blood occupying most of the abdomen; in half gravid, blood occupied only 3–4 segments of the ventral surface and 6–7 segments of the dorsal surface of the abdomen; in gravid females, most blood was digested and the abdomen was whitish and distended. Subsequently, the mosquitoes were taken to the laboratory for morphological identification using keys by Gilles and de Meillon [15]. *An. gambiae s.l.* adults were preserved dried in Eppendorf tubes containing desiccated silica gel for molecular characterization.

### PCR- Identification of the members of ***an. gambiae*** complex

Extraction method had been extensively discussed [16].

### Map processes

A scanned administrative map (1:500.000) of the State was geo-referenced and digitized using Arc View GIS software (version 3.29 ESRI CA, USA) [17,18]. Separate layers were created for the *Plasmodium falciparum* malaria prevalence rates and PCR- identified *An. gambiae* complex from each site. Spatial maps were displayed and classified using a specific identifier of Arc View Spatial Extension [13].

### Spatial analyses

The cell count and K-nearest neighbor analyses in Dave and Uriel [19] were adapted to describe the spatial distribution patterns. The, Mean Variance Ratio (MVA) and near-neighbor (Rn) values were calculated. When MVA or Rn was <1, the spatial pattern is described as clustered (aggregated); when they are equal to 1, the spatial pattern is random and when it is >1, the spatial pattern is even (uniform).

## Results

Five culicid species (*Culex quinquefasciatus* (46.5%), *Anopheles gambiae s.l.* (31.3%), *Aedes aegypti* (13.8%), *Anopheles funestus* (6.1%) *Anopheles nili* (2.3%) were identified during the survey. Differences in species abundance were significant (F = 21.64; df = 4, p-value = 0.00, p < 0.05).

### Species composition of *An. gambiae* complex

Three species (*Anopheles gambiae s.s., An. arabiensis, An. melas*) were identified. *An. gambiae s.s.* was dominant across eco-vegetational zones. Detailed data appear in Ebenezer *et al.*[16].

### Spatial distribution of *An. gambiae* complex and *P. falciparum*

**Figure 1:**
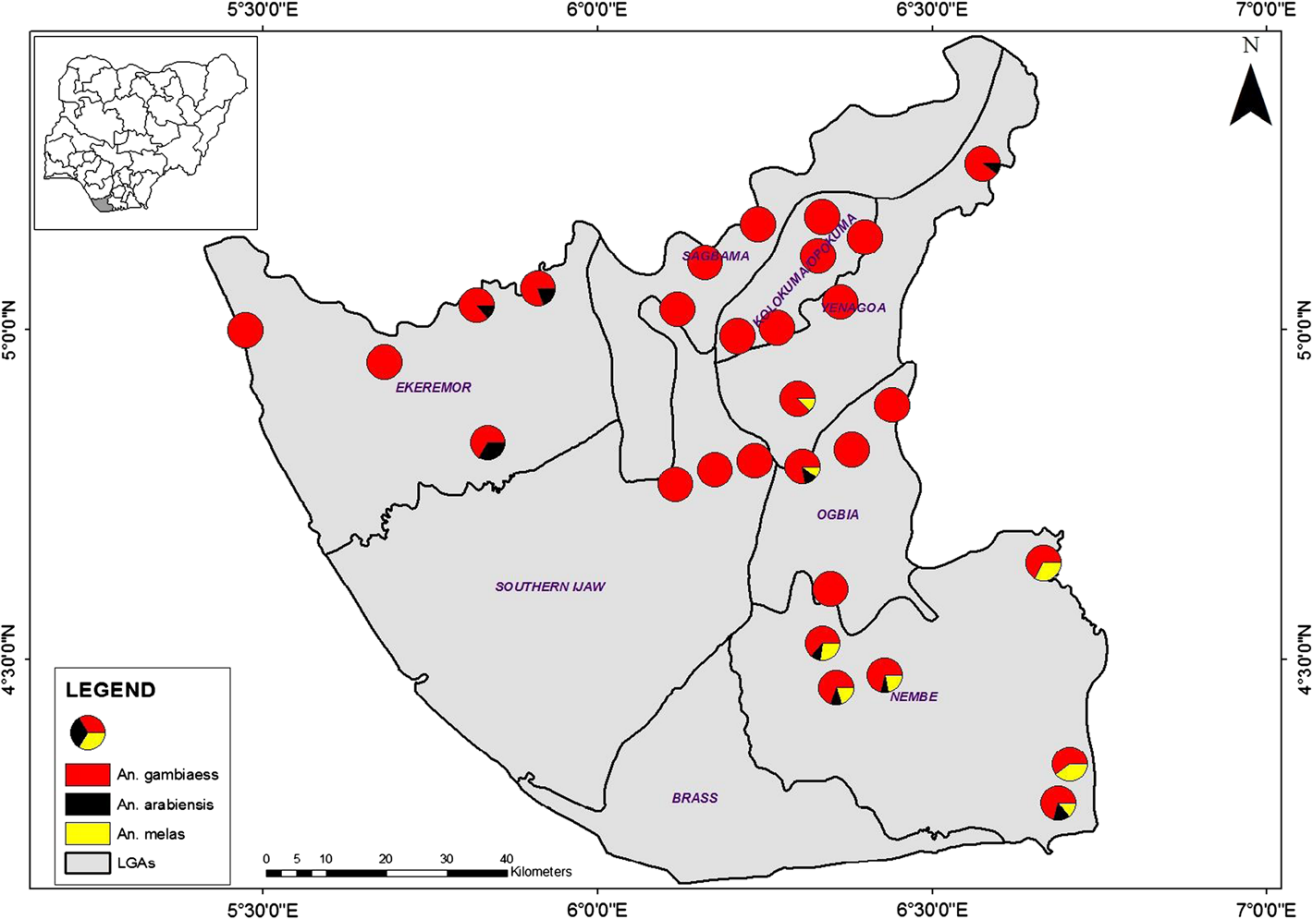
**Spatial distribution of *Anopheles gambiae *** **sibling species across LGAs.**

**Figure 2:**
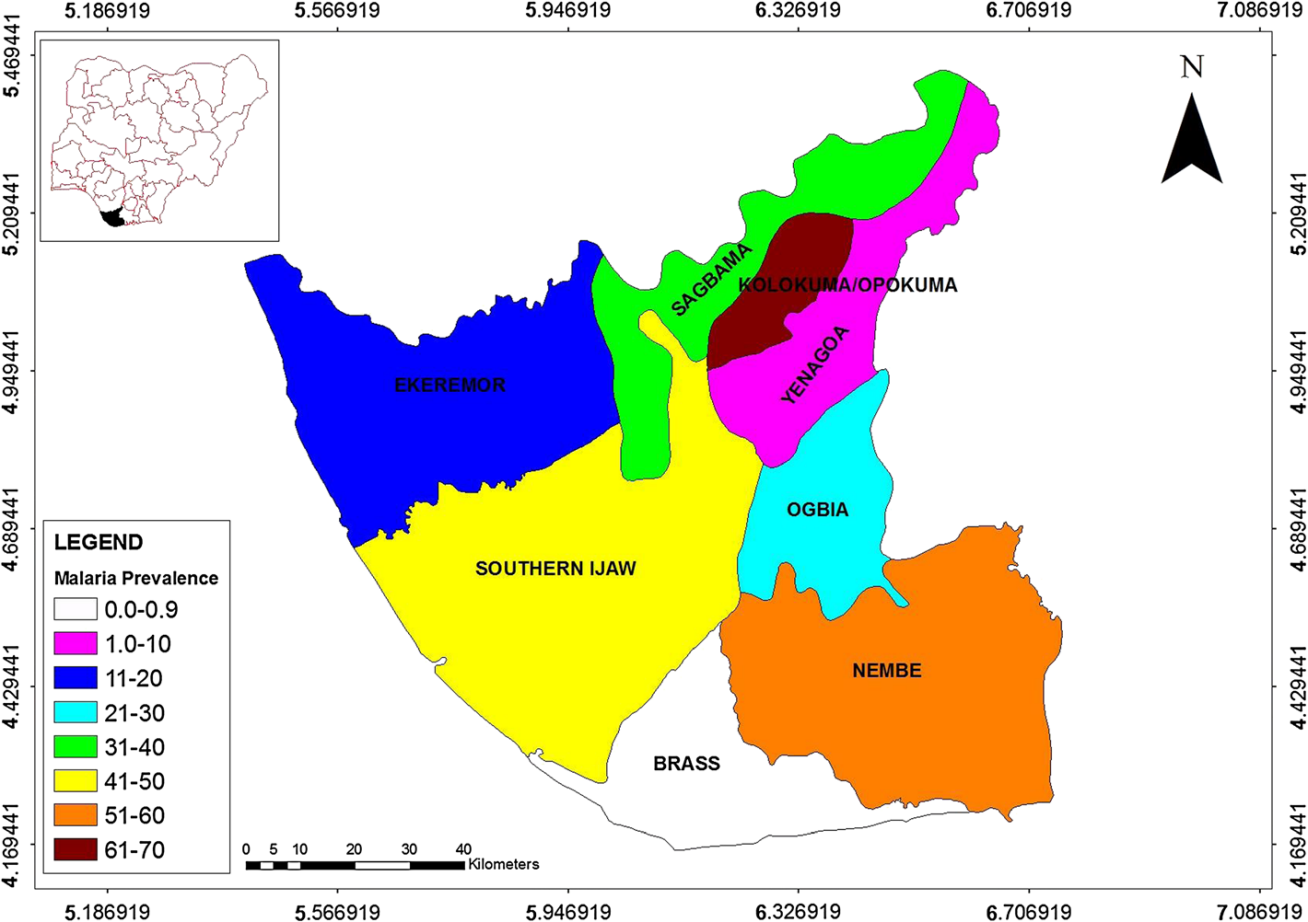
**Spatial distribution of *Falciparum *- malaria prevalence rates across LGAS.**

The spatial patterns of *An. gambiae* sibling species across the study locations showed a tendency to clustering or aggregation (MVA = 0.57, Rn = 0.57) (Table 1). *An. gambiae s.s.* and *An. arabiensis* were sympatric in Ekeremor, while the 3 species (including *An. melas*) were sympatric in Nembe (Figure 1). The gonotrophic (physiological) status examination revealed that 34.3, 23.5, 23.1 and 18.4% of the population were fed, unfed, gravid and half gravid respectively. The highest malaria prevalence rates were obtained at Kolokuma-Opokuma and Nembe LGAs and least in Yenagoa LGA (Table 2). Variation in prevalence rates among the LGAs was significant (t = 5.976, df = 6, p-value = 0.002, p < 0.05). Classifications of the *P. falciparum*-malaria endemicity were: Hypoendemic (PR ≤ 10 < 40%) in Yenagoa, Ekeremor Sagbama and Ogbia LGAs; Mesoendemic (PR ≥ 40% ≤50%) in Southern Ijaw LGA and Hyperendemic (PR > 50%) in Nembe and Kolokuma-Opokuma LGAs (Figure 2). The highest prevalence rate was recorded in the 30-39 yrs age group, and the lowest prevalence was recorded in the 0-9 yrs group (Table 3).

**Table 1:** Summary of nearest neighbour analysis

**Variables**	**Mean distance (D) in km**	**Number of points (N)**	**Total distance**	**Nearest neighbour**
*Anopheles gambiae* sibling species	8.415	32	269.28	0.57*

* Rn is near cluster.

**Table 2:** Malaria prevalence rates in study LGAs

**Eco- vegetational zones**	**LGAs**	**No. of humans examined**	**No. positive**	**% positive**
Fresh water	Yenagoa	1980	245	12.4
	Sagbama	575	159	27.7
	Kolokuma	602	409	69.9
Brackish water	Ogbia	967	215	22.2
	Southern Ijaw	600	169	28.2
	Ekeremor	988	177	18.5
Mangrove forest	Nembe	609	322	52.9
** Total**		**6321**	**1696**	**33.0**

**Table 3:** Age-specific prevalence rates of malaria infection, 2008-2010

**Age range (Yrs)**	**No of persons examined**	**No. positive**	**% positive**
0-9	865	100	11.6
10-19	1053	296	28.1
20-29	1588	264	16.6
30-39	1333	646	48.5
40-49	714	228	31.9
50-59	469	134	28.6
≥60	299	28	9.4
Total	6321	1696	27.4

## Discussion

The clustered spatial patterns among the *An gambiae* sibling species were similar to results obtained by Sogoba *et al.*[20] and probably reflected variation in the favorability of the environment [21]. Sympatric occurrence of the *An. gambiae* complex had been documented [5]. These clusters may serve as nidi of transmission; they may also serve as refugia, where pathogens, vectors and hosts persist during unfavorable periods [22]. The *Anopheles gambiae s.l.* population was virile, with approximately 35% fed and about 40% half gravid or gravid.

Breman [23] provided a list of intrinsic and extrinsic determinants of the malaria burden. The intrinsic factors include: host genetic susceptibility and host immunological status. The extrinsic factors are: parasite species, mosquito species and environmental conditions. Environmental conditions are climatic conditions, and availability of breeding sites. The socio-economic component consists of education, social, behavioural, political and economic status of host populations. Parasite and host populations were apparently not responsible for the variation in malaria prevalence across the State. There were no differences in species of parasites. It was unlikely that genetic susceptibility and immunological status of human hosts varied significantly across the ecovegetational zones and the semi urban/rural divide in the State. Warm temperature, high rainfall and humidity were pervasive across the State; breeding sites were also available because adults were collected throughout the year.

The low malaria prevalence rates in Yenagoa LGA, the State capital and the only semi-urban location in the study area was probably due to the higher living standard (better housing, knowledge of disease, community participation in malaria prevention and control) [24,25]. One of the factors that might have contributed to the high prevalence rates in Nembe LGA, might be attributable to the clusters of the 3 sympatric *An. gambiae* sibling species that formed nidi for transmission [22]. The high prevalence rates in Kolokuma-Opokuma, where *An. gambiae s.s.* was abundant and allopatric could be attributed to the high density and vectorial competence of the efficient *An. gambiae s.s.* as a malaria vector [1,6,16,20,26]. This efficiency may also explain the relatively high prevalence rates in Sagbama and in the area of Southern Ijaw LGA, contiguous with Sagbama where *An. gambiae s.s.* was allopatric. Although the literature indicates that annual deaths from malaria are mainly in infants and young children [27,28]; these results show the highest prevalence rate in the 30-39 yrs age group. It is apparent that the source and composition of sample populations have a significant impact on the pattern of malaria prevalence rates across age groups.

## Conclusion

High malaria prevalence rates were associated with *An. gambiae s.s.* either in allopatry or sympatry across eco-vegetational zones. In areas where the sibling species clustered, they probably formed nidi for transmission. Socio-economic conditions might have contributed to reduced transmission in Yenagoa, State Capital.

## Competing interests

The authors declare that they have no competing interests.

## Authors’ contributions

AE was the doctoral student responsible for the fieldwork and laboratory analyses. MAEN designed the molecular component of the doctoral proposal and supervised the morphological identification of *Anopheles gambiae s.l.* PIA was the Co-Supervisor of AE’s doctoral project. SNO was the Principal Supervisor of AE’s doctoral project and corresponding author. TC facilitated field collections at the communities in Bayelsa State. All authors read and approved the final version of the manuscript.
